# Real-time feedback, debriefing, and retraining system of cardiopulmonary resuscitation for out-of-hospital cardiac arrests: a study protocol for a cluster parallel-group randomized controlled trial

**DOI:** 10.1186/s13063-018-2852-8

**Published:** 2018-09-20

**Authors:** Akihiro Hirakawa, Toshihiro Hatakeyama, Daisuke Kobayashi, Chika Nishiyama, Akiko Kada, Takeyuki Kiguchi, Takashi Kawamura, Taku Iwami

**Affiliations:** 1 Department of Biostatistics and Bioinformatics, Graduate School of Medicine, The University of Tokyo, Tokyo, Japan; 2 Department of Clinical Trials and Research, Clinical Research Center, National Hospital Organization Nagoya Medical Center, Nagoya, Japan; 3 Kyoto University Health Service, Yoshida-Honmachi, Sakyo-ku, Kyoto, 606-8501 Japan; 4 Department of Emergency and Critical Care Medicine, Emergency and Critical Care Center, Dokkyo Medical University Saitama Medical Center, Koshigaya City, Japan; 5 Department of Critical Care Nursing, Kyoto University Graduate School of Human Health Science, Kyoto, Japan

**Keywords:** Bystander, Cardiac arrest, Cardiopulmonary resuscitation, Critical illness, Emergency medical dispatcher, Emergency services, Epidemiology, Resuscitation, Survival

## Abstract

**Background:**

The quality of cardiopulmonary resuscitation (CPR) performed by emergency medical services (EMS) personnel affects patient outcomes after cardiac arrest. A CPR feedback device with an accelerometer mounted on a defibrillator can monitor the motion of the patient’s sternum to display and record CPR quality in real time. To evaluate the utility of real-time feedback, debriefing, and retraining using a CPR feedback device outside of the hospital, an open-label, cluster randomized controlled trial will be conducted in five municipalities of Osaka Prefecture, Japan.

**Methods:**

Each EMS station within a fire department will be randomly assigned to: 1) the treatment group with real-time feedback, debriefing, and retraining using the CPR feedback device (intervention group); or 2) the conventional treatment group without real-time feedback, debriefing, and retraining (control group). This trial will include 2850 to 3020 patients over about 4 years. The primary outcome of the trial is 1-month favorable neurological survival, defined as cerebral performance category scale score 1 or 2. Secondary outcomes are 1-month survival, survival to hospital discharge, return of spontaneous circulation, and quality of CPR including fraction, depth, tempo, and ventilation rate.

**Discussion:**

The trial will assess whether treatment monitored by the CPR feedback device, which allows for real-time feedback, debriefing, and retraining using CPR quality data, outperforms conventional treatment without real-time feedback, debriefing, and retraining in terms of 1-month favorable neurological survival in cardiac arrest patients receiving CPR outside the hospital.

**Trial registration:**

University Hospital Medical Information Network (UMIN) Clinical Trials Registry, UMIN000021431. Registered on 11 March 2016.

**Electronic supplementary material:**

The online version of this article (10.1186/s13063-018-2852-8) contains supplementary material, which is available to authorized users.

## Background

Out-of-hospital cardiac arrest (OHCA) is one of the major public health issues in the developed world [1–5], and more than 100,000 cases occur every year in Japan [6]. The burden of OHCA is also substantial, with estimated incidences of 110 per 100,000 population in the United States and 84 per 100,000 population in Europe [7, 8]. However, despite recurrent updates of the guidelines for cardiopulmonary resuscitation (CPR) and the spread of the “chain-of-survival”, many areas including the United States, Europe, and Japan have suboptimal survival rates of about 10% [6, 7]. The main reasons for this suboptimal survival rate are the low proportion of performed bystander CPR/automatic external defibrillator (AED) use and poor quality of CPR, including that by emergency medical services (EMS) providers as reported by previous research [9, 10]. Preclinical and clinical evidence has demonstrated that high-quality CPR is required to improve outcomes after OHCA [11, 12].

It is well known that the quality of CPR, including the fraction, depth, tempo, and recoil of chest compressions, affects the outcome of cardiac arrest according to the 2015 International Consensus on CPR and Emergency Cardiovascular Care Science with Treatment Recommendations [1–5, 11, 12]. The recommendations encourage that chest compression fraction (CCF) should be more than 60%, the chest compression depth should be 5–6 cm, and the chest compression tempo should be 100–120 per min. CCF means the proportion of actual performed CPR in the resuscitation period. In a study evaluating the relationship between the CCF performed by EMS personnel and survival after OHCA, the proportion of survival to discharge was tripled for CCF of 61–80% compared with CCF of 0–20% [13]. Surprisingly, even professionals such as EMS personnel usually perform suboptimal CPR in real settings [9, 10]. Thus, high-quality CPR is critical to improving survival after cardiac arrest.

The CPR feedback device to be used in this study is mounted on the defibrillator and is equipped with an accelerometer to monitor the motion of the patient’s sternum and indicate CPR quality on the scene [14]. Previous studies, including a large randomized controlled trial (RCT) in real situations [15], have demonstrated that real-time functions such as fraction, depth, tempo, and recoil of chest compressions can significantly improve these the CPR quality of rescuers but failed to show improvements in patient survival [16, 17]. However, the CPR feedback device also allows EMS personnel to review recorded CPR quality that is evaluated based on the quantified fraction, depth, and tempo for use in debriefing and physical retraining after the event. It has been shown that debriefing using data on CPR quality that is recorded by a CPR feedback device significantly improves the quality of CPR [18, 19]. However, there is no evidence whether the combination of real-time feedback, debriefing, and retraining using such a CPR feedback device can increase patient survival after OHCA. The results of a single-arm trial comparing CPR quality and survival before and after the introduction of real-time feedback and retraining using a CPR feedback device suggest positive effects [16]. However, this positive effect might result from some other important factors, not only real-time feedback and retraining. The transportation protocol for patients with cardiac arrest has changed during this trial period. The improvements in post-arrest critical care, such as therapeutic hypothermia and percutaneous coronary interventions, should be considered during this period.

In this cluster RCT, we will examine whether the combination of real-time feedback, debriefing, and retraining using a CPR feedback device will outperform conventional treatment without any feedback, debriefing, and retraining in terms of favorable neurological survival among cardiac arrest patients who receive CPR by EMS personnel. This trial will evaluate the contribution made by the use of a portable defibrillator, in conjunction with debriefing and retraining, to OHCA patient survival. To our knowledge, this is the first cluster RCT incorporating sample size re-estimation, which is a form of adaptive design, to evaluate the utility of real-time feedback, debriefing, and retraining using a CPR feedback device outside of the hospital in EMS personnel. We believe this trial will contribute to facilitating subsequent clinical trials that evaluate medical devices using re-estimation, based on primary outcomes in intervention and control groups. The purpose of this study is to demonstrate a superiority of the intervention group with real-time feedback, debriefing, and retraining using the CPR feedback device over the control group without real-time feedback, debriefing, and retraining with respect to the primary outcome of 1-month favorable neurological survival in patients with cardiac arrest.

## Methods/design

### Study design and settings

This study is designed as a cluster parallel group RCT to demonstrate the superiority of the intervention group with real-time feedback, debriefing, and retraining using a CPR feedback device over a control group and is to be conducted at three fire departments with 32 EMS stations, covering five municipalities in Osaka Prefecture, Japan: Hirakata City, Neyagawa City, Suita City, Minoh City, and Toyono Town. The coordinating office is located at Kyoto University Health Service in Kyoto City, Japan. The randomization ratio is set at 1:1.

### The EMS system in Osaka prefecture and target population

As described in some studies [20–23], the EMS systems in Japan, including those in Osaka Prefecture, are operated by local fire departments. The free emergency telephone number “119” is used nationwide to call for ambulance vehicles. When called, an ambulance is dispatched from the nearest fire station. Each ambulance has three EMS providers including at least one emergency life-saving technician (ELST) who has advanced training in providing pre-hospital emergency care. ELSTs are authorized to insert an intravenous line and an adjunct airway, including supraglottic airway devices, and to use a semi-automatic defibrillator for OHCA patients. Specially trained ELSTs are permitted to insert tracheal tubes and administer intravenous adrenaline. The use of an AED by lay people in Japan was legally approved in July 2004. All EMS providers in Japan are required to perform CPR according to Japanese CPR guidelines and are trained in performing basic resuscitation practices for an OHCA event.

#### Inclusion criteria

All medical etiologies related to OHCA in patients aged 18 years or older who receive CPR by EMS personnel will be included in this trial. Cardiac arrest will be presumed to be of medical etiology unless it is due to external causes such as a traffic accident, a fall from height, suicide, drowning, asphyxia, poisoning by drugs or gas, or hanging [24–26].

#### Exclusion criteria

We will exclude pregnant women or patients who either personally refuse to take part in the study or have that decision made on their behalf by family members.

### Randomization

**Fig. 1:**
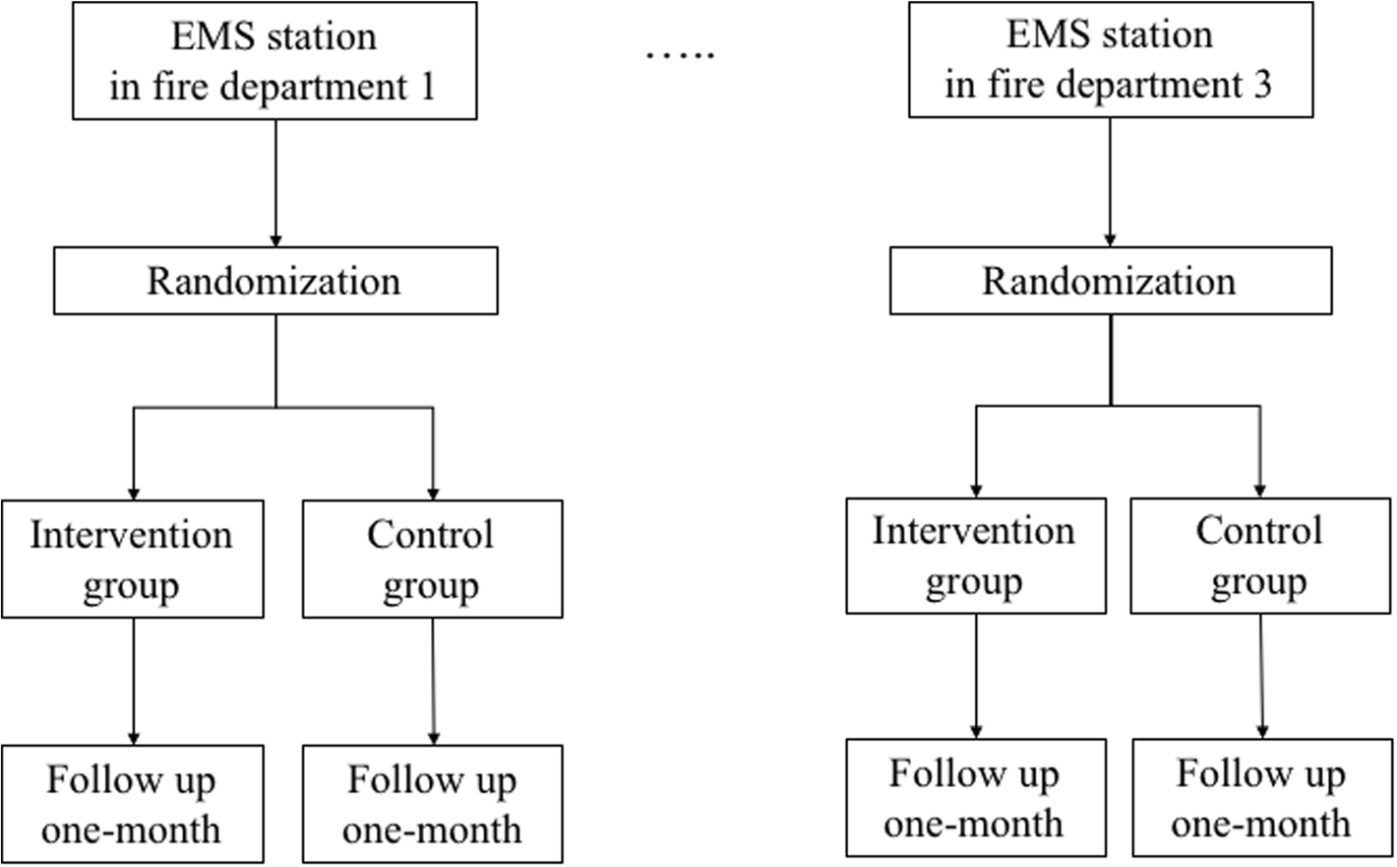
Flowchart of stratified cluster randomization. EMS emergency medical services

The flowchart of randomization is shown in Fig. 1. EMS stations will be randomized in each fire department (cluster randomization) for this trial. A trial statistician, who is independent of the study investigators, will perform permuted block randomization using software to generate random numbers in each stratum. Subsequently, some EMS stations will perform the treatment assigned as the intervention group, and other stations will perform the treatment assigned as the control group throughout the trial. In August 2016, 32 EMS stations in three fire departments will be registered and assigned to the intervention group or the control group. Allocation will not be blinded due to the nature of the intervention. However, to manage the trial, ensure communication with each EMS station, and maintain the function of the CRP feedback device, only selected investigators in the coordinating office will know the allocations. The remaining investigators and trial statisticians will be blinded to the allocation throughout the trial.

### Intervention

**Fig. 2:**
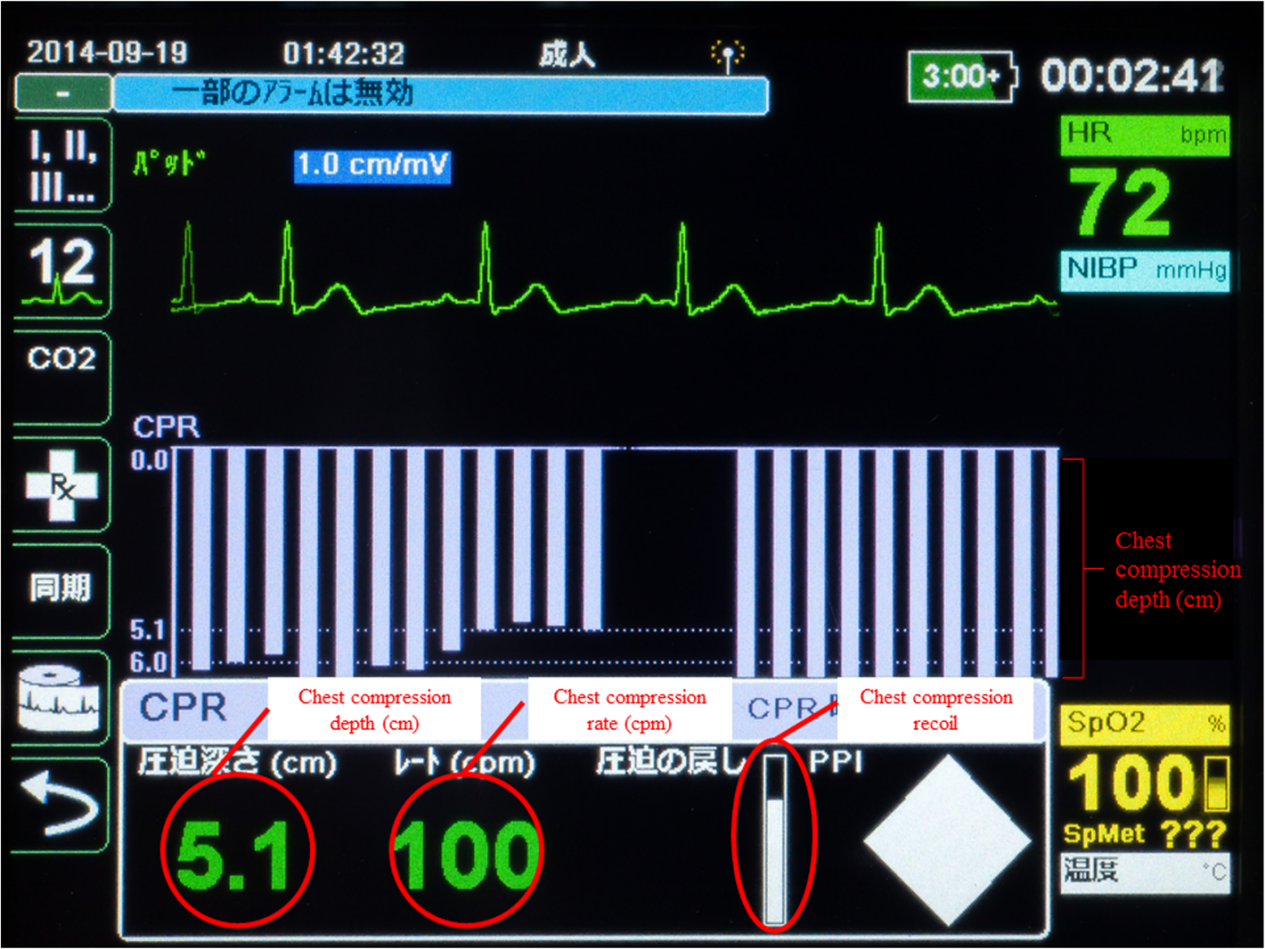
Screen display of the CPR feedback device in the intervention group. Chest compression depth and tempo are presented in cm and cycles per minute (cpm). The whiteness of the bar shows the quality of recoil. The entire bar changes to white upon complete recoil by EMS personnel. CPR cardiopulmonary resuscitation, HR heart rate, NIBP noninvasive blood pressure, PPI perfusion per index, SpO2 blood oxygen saturation, EMS emergency medical services

Firstly, we will recommend that EMS resuscitation time at the scene be more than 10 min for the intervention group. EMS personnel in the intervention group will activate the real-time feedback function of the CPR feedback device on the scene, thereby simultaneously displaying the quality of CPR including depth, tempo, and recoil of chest compressions (Fig. 2) during the resuscitation as measurement items. The manufacturer of the CPR feedback device in this trial is ZOLL Medical Corporation. The real-time feedback is based on the following criteria: 1) chest compression depth of 5–6 cm; 2) chest compressions at a rate of 100–120 per min; 3) immediate recoil to the original position; and 4) minimum interruption of chest compressions. Additionally, hot and cold debriefings will be performed after the event in the intervention group. EMS personnel will review the events using specific software on the day of the event (hot debriefing). Concomitantly, the resuscitation procedure will be evaluated by reviewing the data on CPR quality recorded by the CPR feedback device. Similarly, EMS personnel in the intervention group will review the events using debriefing reports, which will be generated by the data manager at Kyoto University Health Service within approximately 2 weeks after each event (cold debriefing). Using the CPR quality data in cold debriefings, the criteria used to determine whether EMS personnel require retraining are as follows: 1) for a CCF more than 80%, the chest compression depth should be 5–6 cm and the chest compression rate should be 100–120 per min; 2) the average chest compression depth should be 5–6 cm; 3) the average chest compression rate should be 100–120 per min; 4) the ventilation rate after advanced airway management should be 6–10 per min; and 5) peri-shock pause time should be within 10 s. ELST defibrillator use will be one of the criteria. Peri-shock pause time is defined as the total of the pre- and post-shock pause time. Pre-shock pause time is defined as the time interval between chest compression cessation and defibrillator shock delivery. Post-shock pause time is defined as the time between defibrillator shock delivery and chest compression resumption [27]. As facilitators of the intervention group, the captains of the respective EMS stations will evaluate the resuscitation procedures using a checklist during cold debriefings. If the CPR process does not meet the above criteria, EMS personnel will be required to receive physical retraining after watching an educational video about the real-time feedback system. Physical training scenarios are focused on unpassed point of criteria. These scenarios are produced by facilitators. Cases to be considered for the physical retraining program will be those not meeting the criteria in real situations. Training in the program emphasizes the “team approach to resuscitation”. EMS personnel may complete the training program an unlimited number of times. The educational video emphasizes watching the screen of the CPR feedback device during the resuscitation to realize the quality of CPR in real time. Additionally, the video shows sophisticated CPR performance by EMS personnel. To introduce this physical retraining program, we evaluated the feasibility of the program on 27 July 2016 at Kyoto Tachibana University before starting the trial.

In the control group, EMS personnel will perform resuscitation without real-time feedback, hot and cold debriefings, or physical retraining using the CPR feedback device. However, EMS personnel in the control group will use the CPR feedback device as a simple defibrillator without activating the real-time feedback function on scene during this trial period.

All EMS providers in Japan, including the intervention group and the control group in this trial, must perform resuscitation practices according to Japanese CPR guidelines based on the 2015 International Consensus on CPR and Emergency Cardiovascular Care Science with Treatment Recommendations.

### Outcomes

The primary outcome is 1-month neurological survival of OHCA patients, measured using the cerebral performance category (CPC) score. CPC 1 indicates good cerebral performance, CPC 2 indicates moderate cerebral disability, CPC 3 indicates severe cerebral disability, CPC 4 indicates coma or vegetative state, and CPC 5 indicates death (Table 1). We define CPC 1 or 2 as favorable neurological survival in cardiac arrest patients. These categories will be clinically judged by the physicians in charge in collaboration with EMS personnel [24–26]. The background of the physicians in charge is generally that of emergency medicine. All patients who survive the cardiac arrest will be followed for up to 1 month after the event by the EMS providers in charge. Neurologic outcome using CPC score will be confirmed by means of a follow-up interview 1 month after successful resuscitation. The main reason for discharging from hospital before 1 month after the event is in-hospital death. The patients who survive the OHCA receive rehabilitation therapy in hospital. Therefore, specialized evaluation procedures for patients who are discharged from hospital before 1 month after the event are not required. The secondary outcomes are as follows: 1-month survival; survival to hospital discharge; return of spontaneous circulation; and quality of CPR including chest compression depth, chest compression tempo, CCF, and ventilation rate.

**Table 1:** Cerebral performance category score

1 Good cerebral performance	
2 Moderate cerebral disability	
3 Severe cerebral disability	
4 Coma or vegetative state	
5 Death	

### Data collection

Data will be collected using international Utstein-style guidelines on reports for OHCA and using the CPR feedback device with an automatic acceleration sensor [14, 24–26]. The data are checked for consistency by the computer system and are confirmed by the study investigators. If the data form is incomplete, the researchers will return it to each EMS station and the form will be completed. EMS personnel check the quality of the defibrillator every day. Additionally, the CPR feedback device will be calibrated by the manufacturer once per year.

### Statistical analyses

All statistical analyses will be performed based on the intent-to-treat principle. Patient backgrounds will be summarized using means and standard deviations for continuous variables and numbers and proportions for categorical variables. The effectiveness of the feedback device will be calculated using a generalized mixed-effect model with exchangeable covariance matrix. The model includes the treatment arm (intervention vs. control), three departments (Hirakata City, Neyagawa City, Suita City, Minoh City, and Toyono Town), patient sex (female vs. male), patient age (< 65 years vs. ≥ 65 years), first recorded cardiac rhythm (shockable vs. non-shockable), site of cardiac arrest (home vs. other), witnessing of collapse (yes vs. no), bystander CPR (no CPR performed vs. compression-only CPR vs. conventional CPR with rescue breathing), time to arrival of EMS personnel at the scene, and professional experience (years) of EMS providers. These analyses will also be performed for respective subgroups of witnessing cardiac arrest, site of cardiac arrest, and starting time of CPR from cardiac arrest. All statistical tests will be two-sided, and *P* < 0.05 will be considered statistically significant. All analyses will be performed using SAS version 9.4 (SAS Institute, Cary, NC, USA). Reporting of the results will follow the CONSORT guidelines for reporting of cluster randomized trials [28]. All plans for statistical analyses will be reviewed and approved by an independent statistician of the Data Monitoring Committee (DMC).

### Determination of sample size

Based on previous studies [16–19], the odds ratio of the intervention group relative to the control group with respect to 1-month favorable neurological survival is expected to be 1.65. The required number of patients in the intervention and the control groups for a given number of participating EMS stations under a two-sided significance level of 5% and statistical power of 80% are shown in Table 2. We started the trial at 32 EMS stations and will add a new EMS station up to 50 stations during the trial to accelerate the patient enrollment; therefore, the total sample size will range from 2850 to 3020.

**Table 2:** Required sample size according to the number of EMS stations

Number of EMS stations	Number of patients enrolled per each EMS station (cluster size)	Total sample size
20	151	3020
30	97	2910
40	72	2880
50	57	2850

EMS emergency medical services

In RCTs, it is desirable to have a sufficient sample size to achieve a desired statistical power of testing for detecting a clinically meaningful difference. In our cluster RCT, the sample size was determined using the initial guess value of odds ratio of the intervention group relative to the control group for 1-month favorable neurological survival. However, if this value is not true, the trial may not achieve the desired power. One idea to address this concern is to re-estimate the required sample size based on the observed odds ratio at an interim analysis during the trial. Thus, we incorporated sample size re-estimation as a form of adaptive design.

### Interim analysis and sample size re-estimation

The interim analysis will be performed upon completion of the primary endpoint assessment for 1500 patients to determine whether a re-estimation of the total is required; this will be performed by the independent DMC, consisting of two physicians and a statistician. Sample size re-estimation will be planned using the method proposed by Mehta and Pocock [29]. Specifically, at the time of interim analysis, the conditional power for detecting the difference in primary outcome between the two groups in the final analysis will be estimated using the accumulated data of 1500 patients. When the conditional power is between 50% and 80%, we will increase the sample size accordingly, up to a sample size of 5040. Otherwise, the trial will continue using the planned sample size.

### Trial monitoring

Monitors in the coordinating office will track the data on CPR quality and register any deviation found in the device during the trial. Serious adverse events and serious adverse device actions will be reported to the coordinating office upon occurrence.

### Schedule of data collection

**Fig. 3:**
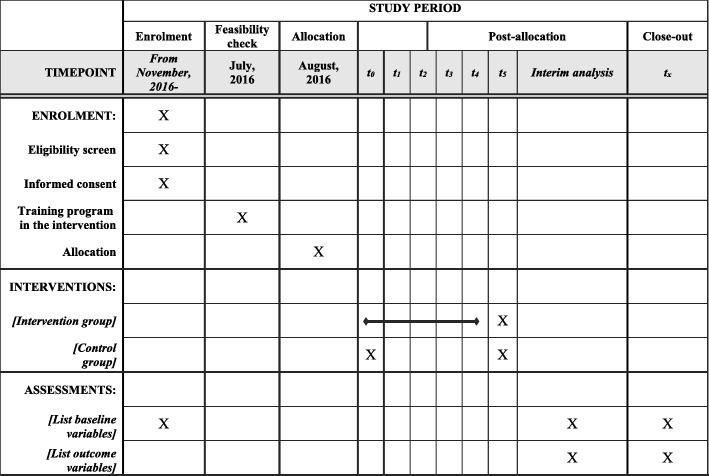
Trial schedule of enrolment, interventions, and assessments. *T*_*0*_ means performing resuscitation using the CPR feedback device, *t*_1_ real-time feedback, *t*_2_ hot debriefing, *t*_3_ cold debriefing, *t*_4_ physical retraining, *t*_5_ data collection on resuscitation practices and patient outcomes. The interim analysis will be performed upon completion of the primary endpoint assessment for 1,500 patients to determine whether a re-estimation of the total is required; this will be performed by the independent Data Monitoring Committee, consisting of two physicians and a statistician. CPR cardiopulmonary resuscitation

Treatment assignment will be performed before starting the trial. Patients will be followed up for 1 month after enrollment. The schedule for data collection is summarized in Table 3 and Fig. 3.

**Table 3:** Treatment assignment and data collection

	Assessments and procedures	Before starting trial	Enrollment (location of CPR)	During CPR	Hospitalization
Fire department	Treatment assigned	X			
Emergency ambulance crew profile	Experience (years)	X^a^			
	Qualification as emergency medical technician	X^a^			
Patient profile	Informed consent		X^b^	X^b^	X ^b^
	Sex				X
	Age				X
	Witnessing cardiac arrest				X
	Site of cardiac arrest				X
	Bystander CPR				X
	Initial electrocardiogram waveform				X
	Survival confirmation				X
	Discharge from hospital				X
	CPC				X
Quality of CPR	Chest compression fraction			Automatically collected during resuscitation	
	Depth and speed of chest compressions				
	Ventilation-related data				

*CPC* cerebral performance category, *CPR* cardiopulmonary resuscitation

^a^Collected every year

^b^If possible

### Approvals

This trial will be conducted in accordance with the Declaration of Helsinki, the World Medical Association, and the Ethical Guidelines for Medical and Health Research Involving Human Subjects of Japan. The requirement for individual EMS informed consent has been waived because this study will be performed in a work environment setting. This trial is registered in the University Hospital Medical Information Network (UMIN) Clinical Trials Registry under number UMIN000021431 (https://upload.umin.ac.jp/cgi-open-bin/ctr_e/ctr_view.cgi?recptno=R000024721). If a family member of a patient is available at the scene and is in stable condition, EMS personnel will inform them about the trial using a handout, either at the scene or in the ambulance, and thus attempt to obtain verbal consent on the patient’s behalf. However, after the patient regains consciousness, EMS personnel will try to obtain verbal informed consent from the patient. We have provided information concerning this study on our research group’s website (http://cc-resus.com/research/files/2017/03/001.pdf, in Japanese), including the right to refuse use of personal data for the trial. The protocol of this trial has been approved by all participating fire departments as well as the ethics committee of Kyoto University Graduate School of Medicine (registration number C-1154 Additional file 1).

## Discussion

We will re-estimate the sample size considering the actual incidence of 1-month favorable neurological survival in the intervention group and the control group at the interim analysis. This is the first RCT to perform sample size re-estimation with an interim analysis by considering the difference in primary outcomes between an intervention group and control group to evaluate a medical device rather than a drug. We will report the results and findings in this trial through peer-reviewed papers.

Previous studies have shown that sophisticated CPR quality is essential to improve outcome after OHCA [30, 31]. However, EMS personnel generally provide poor quality of CPR to patients at the cardiac arrest scene. The main content of intervention in this study is to improve performed CPR quality by EMS personnel through real-time feedback, debriefing, and retraining using a CPR feedback device. If these interventions could improve the quality of CPR, the findings of this study would contribute to increased favorable neurological survival after sudden cardiac arrest.

This study will use data recorded by a specific CPR feedback device; therefore, the study findings will not be applicable to EMS systems that do not use this device. Additionally, the findings of our trial may not be thoroughly generalizable to other districts because this study was conducted in a single prefecture.

### Trial status

This trial is on-going and patient recruitment began in February 2017. The recruitment is not completed at the time of submission and will finish when 2850 to 3020 participants have been included.

## Additional file

Additional file 1: SPIRIT 2013 Checklist: Recommended items to address in a clinical trial protocol and related documents. (It is strongly recommended that this checklist be read in conjunction with the SPIRIT 2013 Explanation & Elaboration for important clarification on the items. Amendments to the protocol should be tracked and dated. The SPIRIT checklist is copyrighted by the SPIRIT Group under the Creative Commons “Attribution-NonCommercial-NoDerivs 3.0 Unported” license2) (DOC 126 kb)

## Abbreviations

AED: Automatic external defibrillator
CCF: Chest compression fraction
CPC: Cerebral performance category
CPR: Cardiopulmonary resuscitation
DMC: Data Monitoring Committee
ELST: Emergency life-saving technician
EMS: Emergency medical services
OHCA: Out-of-hospital cardiac arrest
RCT: Randomized controlled trial
UMIN: University Hospital Medical Information Network
